# 372. Impact of Updated 2020 Public Health Service Guidelines on Deceased Organ Donors and Transplant Recipients

**DOI:** 10.1093/ofid/ofae631.113

**Published:** 2025-01-29

**Authors:** Dzhuliyana Handarova, Jesse Howell, Lara A Danziger-Isakov, Stephanie M Pouch

**Affiliations:** United Network for Organ Sharing, Richmond, VA; United Network for Organ Sharing, Richmond, VA; Cincinnati Children's Hospital, Cincinnati, Ohio; Emory University School of Medicine, Atlanta, GA

## Abstract

**Background:**

New guidelines for assessing solid organ donors and recipients for Human Immunodeficiency Virus (HIV), Hepatitis B (HBV), and Hepatitis C (HCV) infection were published by the US Public Health Service (PHS) in 2020 and implemented into Organ Procurement and Transplantation Network (OPTN) policy on 3/1/21. Changes included a shorter risk criteria inclusionary timeframe and the removal of 4 risk criteria.

**Figure:**
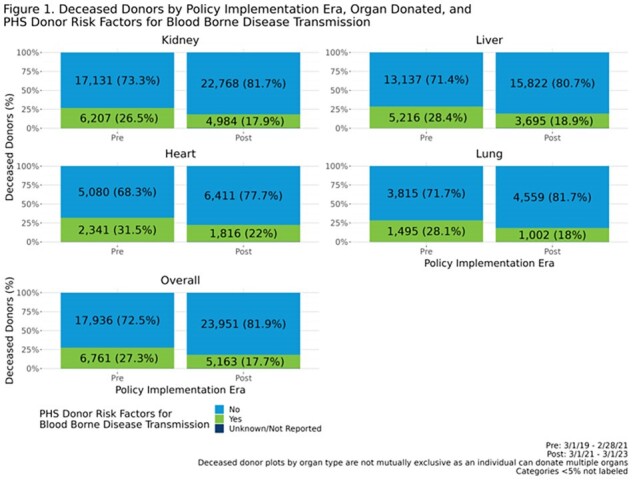

**Methods:**

We analyzed OPTN deceased donor and transplant data 2 years pre-(3/1/19-2/28/21) and post-(3/1/21-3/1/23) implementation of the updated guidelines. For Kaplan-Meier survival analyses, the post- cohort included recipients transplanted before 1/1/23 to ensure recipients had at least 1 year follow-up plus a 3-month data lag. Utilization rates, defined as number of organs transplanted divided by number of available organs from donors with at least 1 organ recovered for the purpose of transplant, are shown for thoracic organs. Non-use rates, defined as number of organs recovered for the purpose of transplant but not transplanted divided by the total number of organs recovered for the purpose of transplant, are shown for abdominal organs.

**Figure:**
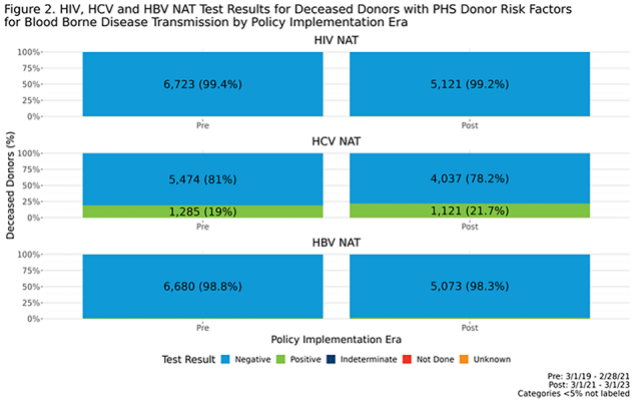

**Results:**

The proportion of donors considered to have PHS risk factors decreased by approximately 10% post-policy, both overall and by organ type (**Fig. 1**). There were no major differences pre- to post- in reported donor HIV, HCV or HBV NAT test results (**Fig2.** ). Post-policy, utilization rates for heart and lung for donors with risk factors were 34.9% and 17.5% respectively, compared to 34.3% and 20.0% pre-policy; while kidney and liver non-use rates for donors with risk factors were 20.3% and 8.2% respectively, compared to 17.2% and 7.9% pre-policy. There was no significant change in 1-year post-transplant patient survival for recipients who received organs from donors with PHS risk factors (**Fig. 3**).

**Figure:**
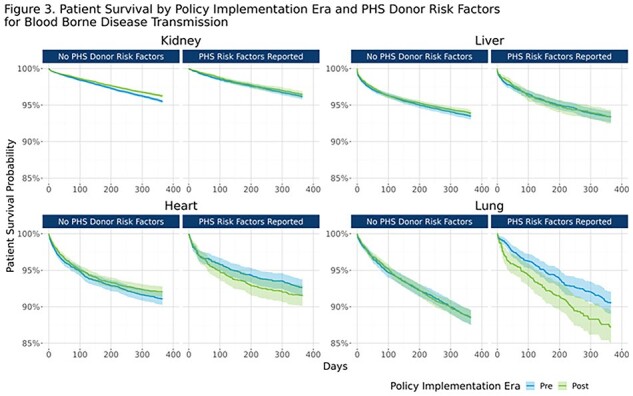

**Conclusion:**

Less restrictive PHS guidelines led to fewer donors being classified as having risk factors for disease transmission. Updated guidelines did not significantly affect donor infectious disease test results or post-transplant survival for recipients. Changes in donor organ utilization and non-use may be due to donor pool changes and/or overlapping policy implementations, and further investigation is warranted.

**Disclosures:**

**Lara A. Danziger-Isakov, MD, MPH**, Aicuris: clinical research contract, paid to institutio|Ansun BioPharma: clinical research contract, paid to institution|Astellas: Advisor/Consultant|Astellas: clinical research contract, paid to institutio|Merck: clinical research contract, paid to institutio|Pfizer: Grant/Research Support|Takeda: clinical research contract, paid to institutio

